# Relationships among bedding materials, bedding bacterial composition and lameness in dairy cows

**DOI:** 10.5713/ajas.20.0565

**Published:** 2020-11-03

**Authors:** Han Li, Xiangming Wang, Yan Wu, Dingran Zhang, Hongyang Xu, Hongrun Xu, Xiaoguang Xing, Zhili Qi

**Affiliations:** 1 Department of Animal Nutrition and Feed Science, College of Animal Science and Technology, Huazhong Agricultural University, Wuhan 430070, China

**Keywords:** Bacterial Composition, Bedding Material, Dairy Cows, Lameness

## Abstract

**Objective:**

Bedding materials directly contact hooves of dairy cows and they may serve as environmental sources of lameness-associated pathogen. However, the specific composition of bacteria hidden in bedding materials is still not clear. The aim of this study was to determine the effect bedding material and its bacterial composition has on lameness of Holstein heifers.

**Methods:**

Forty-eight Holstein heifers with similar body weights were randomly assigned into three groups including sand bedding (SB), concrete floor (CF), and compost bedding (CB). Hock injuries severity and gait performance of dairy cows were scored individually once a week. Blood samples were collected at the end of the experiment and bedding material samples were collected once a week for Illumina sequencing.

**Results:**

The CF increased visible hock injuries severity and serum biomarkers of joint damage in comparison to SB and CB groups. Besides, Illumina sequencing and analysis showed that the bacterial community of CB samples had higher similarity to that of SB samples than CF samples. Bacteria in three bedding materials were dominated by gastrointestinal bacteria and organic matter-degrading bacteria, such as *Actinobacteria*, *Firmicutes*, and norank *JG30-KF-cM45*. Lameness-associated *Spirochaetaceae* and *Treponeme* were only detected in SB and CB samples with a very low relative abundance (0% to 0.08%).

**Conclusion:**

The bacterial communities differed among bedding materials. However, the treponemes pathogens involved in the pathogenesis of lameness may not be a part of microbiota in bedding materials of dairy cows.

## INTRODUCTION

Lameness is one of the most common diseases in the dairy industry that result in increased culling rate and economic losses [1]. The methods for diagnosis and therapy in lameness have greatly improved [2,3]. However, it is also critical to develop preventive measures of lameness in indoor housing systems. To provide clean and comfortable bedding material is one of the key aspects of controlling environmental lameness on farms [4], this benefit is partly due to the animal welfare improvement and reduced exposure to pathogens [5].

Joint damage and digital dermatitis (DD) are the two leading causes of lameness. The prevalence of lameness and severity of joint damage increased in the cows housed on concrete floors (CF), with a higher risk of slipping and higher hoof pressure than comfortable bedding materials [6]. Sand bedding (SB) was one of the most common materials applied on large farms [7]. Besides, compost bedding (CB) has been developed rapidly in housing management [8], those deep-loose bedding materials were often reused by dairy producers for economic profits [4]. Organic bedding material (such as CB and recycled sand) generally harbored a large amount of bacteria, including non-pathogenic bacteria and pathogens [5,7]. Previous studies have reported that DD-associated pathogens might originate from the microbiome of the cow’ oral cavity, gastrointestinal tract, and feces [9,10]. The hooves of dairy cows have direct contact with bedding materials for approximately 12-h per day [11]. Therefore, the bedding materials may serve as a reservoir of DD-associated pathogens. It is important to understand bacterial composition and structure in different bedding materials.

The core bacteria which have been identified in DD lesions are genus *Treponema*, *Mycoplasma*, *Fusobacterium*, *Porphyromonas*, and *Dichelobacter* [12,13]. DD-associated *Treponema* was reported to be present in the housing facility of the dairy herds with clinical DD history [14,15]. However, except for traditional pathogens such as *Treponema*, different bedding materials may selectively promote the survival and growth of other bacterial taxa related to DD lesions. The knowledge of the bacterial composition of the bedding materials in dairy cattle housing is still limited. We assumed that the CB housing system will reduce joint inflammatory response and thus improve behavior performance and joint health. Furthermore, we hypothesized the bacterial composition may be very different between bedding materials. Here, the aim of this study was to determine the diversity and composition of bacteria on different bedding materials (CB, SB, and CF), as well as to evaluate the cows’ comfort and joint damage.

## MATERIALS AND METHODS

### Ethics of statement

The protocol of this experiment was approved by the Scientific Ethic Committee of Huazhong Agricultural University (HZAUCA-2017-011), and the animal trial was conducted in accordance with the National Institute of Health Guidelines for the Care and Use of Experiment Animals (Beijing, China).

### Animals and treatments

A total of 48 healthy Holstein heifers (body weight, 470±23 kg; age, 14 to 16 months) were randomly divided into three groups (16 cows per group). Three independent pens within the same barn were respectively assigned to three bedding treatments including SB, CF, and CB. During the experimental period lasting 50 days, the animals were fed a total mixed ration three times daily (05:00, 12:00, and 17:00) and had free access to feed and water. Each pen contained eating area, drinking area, stall area, and exercise lot. The sand was recycled from the sand separator and then placed in an approximately 70 cm layer on compacted soil. The CB consisted of sawdust, rice straw and organic manure solids, the total height of CB was approximately 70 cm on compacted soil. The sand and CB were plowed and refreshed once a week.

### Hock score and gait score

Hock injuries severity and gait performance of dairy cows in three groups were scored individually once a week by 1 trained observer. The severity of back leg hocks injury was measured using the 6-point scale scoring system as described previously [16]. The gait scoring was evaluated using a 5-point scale scoring system as described previously [17]. An average of 5 measurements on hock score and gait score were respectively calculated for each cow.

### Sample collection

Bedding material samples were collected once a week in different areas of bedding location (eating area, sunny side, night side, lying area, and drinking area). In every area, the samples of SB and CB were collected in the surface, middle layer (10 cm depth) and deep layer (30 cm depth), while the samples of CF were collected on the surface using a medical cotton swab. All samples were placed in 5mL cryopreservation tube and immediately stored at −80°C for further analysis of microbial diversity.

Blood samples were collected through the jugular vein before morning feeding at the end of the experiment, and samples were placed into separation gel coagulation promoting tubes and centrifuged within 2 h at 3,000×g for 15 min to obtain serum. Serum biomarkers of joint damage which include cross linked C-opeptide of type II collagen (CTX-II), procollagen IIA N-terminal peptide (PIIANP) and cartilage oligomeric matrix protein (COMP) were all detected by ELISA kits (JYM0151Bo, JYM0148Bo, JYM0144Bo, ColorfulGene Biotechnology, Wuhan, China).

### Bacterial DNA extraction, PCR amplification, Illumina Misequencing and bioinformatic analysis

The total bacterial genomic DNA of bedding samples was extracted following the instructions of the TIANamp Stool DNA Kit (DP328). Then the quantity of extracted DNAs was examined by NanoDrop ND-1000 spectrophotometer (Thermo Fisher Scientific, Waltham, MA, USA) and the quality was detected by 1% agarose gel electrophoresis. The V3–V4 regions of 16S bacterial rRNA genes were amplified by PCR (ABI GeneAmp 9700, Foster City, CA, USA) using the forward primer 338F (5’-ACTCCTACGGGAGGCA GCA-3’) and the reverse primer 806R (5’-GGACTACHVGG GTWTCTAAT-3’). The relative concentration and fragment length of PCR amplicons in each sample were determined by 2% agarose gel. Then amplicons’ purification was performed by Agencourt AMPure Beads (Beckman Coulter, Indianapolis, IN, USA) and gathered in equal quantity, pair-end 2×250 bp sequencing was performed on an Illumina Misequencing platform.

Operational taxonomic units (OTUs) were clustered from above sequences at 97% similarity. Alpha diversity of bacterial community was indicated by richness estimators and diversity indices based on OTU level. Shared OTUs among three groups were distinguished using Venn. Beta diversity was displayed by principal coordinate analysis (PCOA) and distance heatmap based on weighted UniFrac distances. Correlation analysis between bedding material bacteria genera and serum biomarkers of joint damage were performed using Pearson correlation coefficient on the free online Majorbio I-Sanger Cloud Platform (www.i-sanger.com).

### Statistical analysis

Statistical analysis was performed by using SPSS software (SPSS v. 21, SPSS Inc.; Chicago, IL, USA). Significance analysis based on the hock score, gait score, serum biomarkers of joint damage, alpha diversity, and relative abundances of bacterial communities at different taxonomic levels were conducted by using one-way analysis of variance in SPSS. Statistical significance is defined when p<0.05.

## RESULTS

### Hock and gait scores of dairy cows in different bedding materials

**Figure 1:**
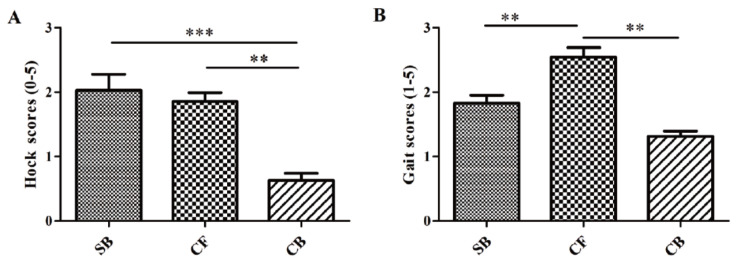
Hock and gait scores of dairy cows housed on different bedding materials. (A) The Hock scores were measured based on a 6-point scale (0–5), and (B) the Gait scores were measured based on a 5-point scale (1–5). SB, sand bedding; CF, concrete floor; CB, composite bedding. Values are presented as mean±standard error of means. and significant differences were displayed with either ** p<0.01, *** p<0.001.

The hock and gait scores were measured based on a 6-point scale (0–5) and 5-point scale (1–5), respectively. A higher score means a worse health condition. The severity of hock lesions injury on CB cows was significantly (p<0.05) lower than SB and CF cows (Figure 1A), the gait score of CF cows was the highest among three groups (Figure 1B).

### Serum biomarkers of joint injuries in different bedding materials

As presented in Table 1, the concentrations of PIIANP and CTX-II in CF group were significantly higher than in SB and CB groups (p<0.05), while no significant difference in COMP level was found among three groups (p<0.05).

### Bacterial Alpha and Beta diversity in different bedding materials

**Figure 2:**
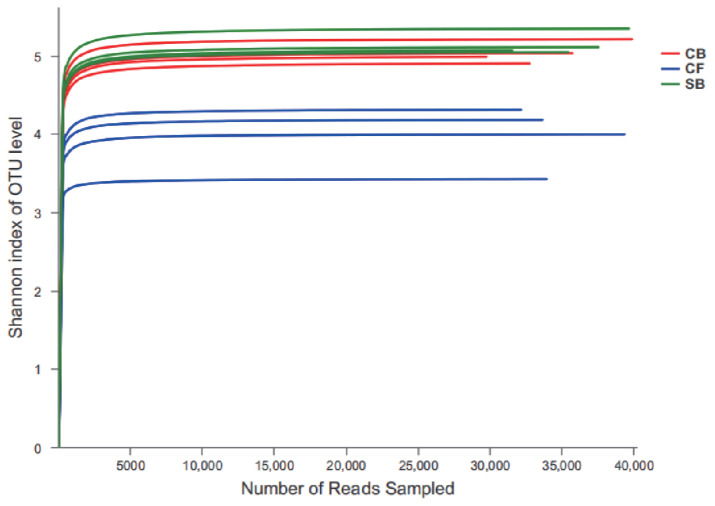
Rarefaction curves based on Shannon index of operational taxonomic units level from bacteria in all the samples. SB, sand bedding; CF, concrete floor; CB, composite bedding. The curves of the same color are different samples within one group (n = 4/group).

The average number of OTUs was the highest in CF group (463.50), followed by SB (900.75), and CB (820.75) groups. However, the CF samples showed significantly lower bacterial diversity (diversity indices: Shannon and Simpson, p<0.05) and richness (richness estimators: Chao and ACE, p<0.05) than other two groups, while the SB group had significantly higher bacterial richness than the CB group (p<0.05). The Good's coverage value in three groups were all more than 0.99 and it implied that the sequencing depth was enough (Table 2). The rarefaction curves based on Shannon index of OTU level tended to be smooth with the increase of sequences number, indicating that collected sequences in each sample were reasonable (Figure 2).

**Figure 3:**
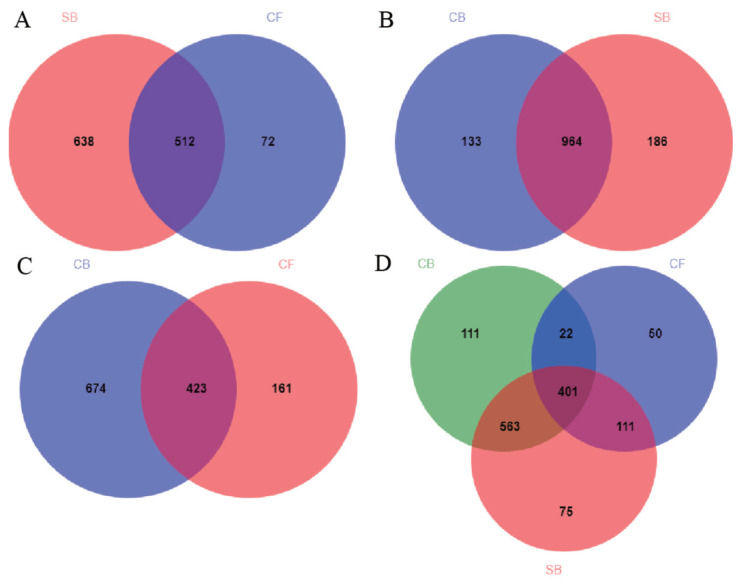
Venn diagram of unique and shared bacterial operational taxonomic units at the 97% similarity level for (A) SB and CF, (B) CB and SB, (C) CB and CF, and (D) SB, CF, and CB. SB, sand bedding; CF, concrete floor; CB, composite bedding.

**Figure 4:**
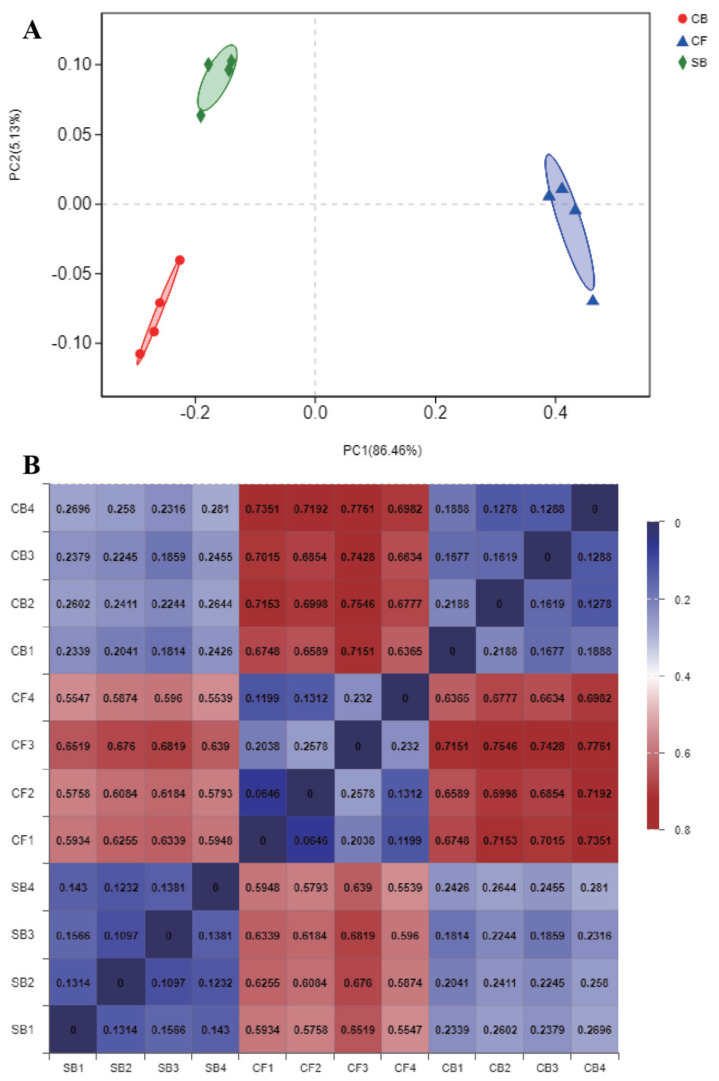
Beta diversity analysis based on weighted Unifrac distance of bacteria in all the samples from different bedding materials. (A) Principal coordinate analysis (PCoA) against PC1 versus PC2 axes. (B) Beta diversity heatmap, the smaller coefficient means higher similarity between two samples. SB, sand bedding; CF, concrete floor; CB, compost bedding.

The Venn diagram visually reflected the core and unique bacterial OTUs among three groups. The number of shared OTUs between SB and CF groups (Figure 3A), SB and CB groups (Figure 3B), CF and CB groups (Figure 3C), and among three groups (Figure 3D) were 510, 963, 420, and 398, respectively. There were 75, 50, and 109 unique OTUs in SB, CF, and CB groups, respectively (Figure 3D). Beta diversity analysis including PCoA and distance heatmap based on weighted Unifrac distance were used to assess variation in bacterial communities’ composition across all samples. The bacterial community in SB group was structurally similar to CB group as revealed by spatial position in PCoA (Figure 4A), this was consistent with beta diversity distance heatmap (Figure 4B), the coefficient between SB and CB samples (0.1814 to 0.2810) was lower than that between SB and CF samples (0.5539 to 0.6819) and that between CF and CB samples (0.6365 to 0.7761) (Figure 4B).

### Dominant bacterial taxa in different bedding materials

**Figure 5:**
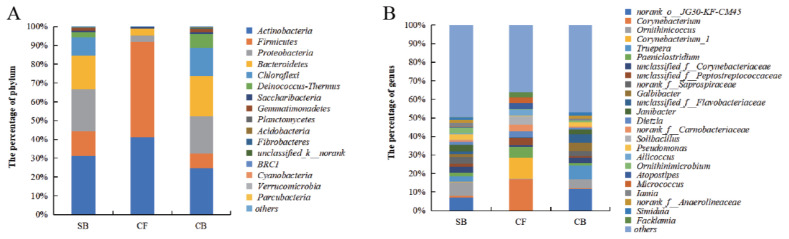
Bacterial community at (A) phylum and (B) genus level of all the samples from different bedding materials. SB, sand bedding; CF, concrete floor; CB, compost bedding.

The obtained OTUs of all samples from the SB, CF, and CB groups could be classified into 26, 11, and 24 phyla, respectively. The dominant phyla were *Actinobacteria*, *Firmicutes*, *Proteobacteria*, *Bacteroidetes*, and *Chloroflexi*, which together accounted for 94.35%, 99.05%, and 88.73% of total relative abundance in SB, CF, and CB groups, respectively (Figure 5A). *Actinobacteria* dominated bacteria communities in SB (31.30%) and CB (24.75%) groups. *Firmicutes* (50.75%) was predominant in CF samples. *Proteobacteria*, *Actinobacteria*, and *Bacteroidetes* were the second most prevalent phyla in SB (22.45%), CF (41.15%), and CB (21.50%) groups, respectively (Figure 5A).

At the family level, *Corynebacteriaceae*, *Intrasporangiaceae*, and *Flavobacteriaceae* were the most abundant families in SB and CB samples, while the dominated bacteria families in CF were *Corynebacteriaceae*, *Peptostreptococcaceae*, *Carnobacteriaceae*, *Staphylococcaceae*, *Ruminococcaceae*, and *Clostridiales Family XI* (Supplementary Table S1).

The dominance of bacterial genera also varied among different bedding materials. The four dominant genera in the SB group were *Ornithinicoccus* (7.38%), norank *JG30-KF-CM45* (7.11%), norank *Saprospiraceae* (3.63%), *Janibacter* (3.43%). The *Corynebacterium* (16.88%), *Corynebacterium_1* (11.33%), *Paeniclostridium* (5.66%), and *Solibacillus* (4.61%) were dominant genera in CF group. In bacterial genera of CB group, norank *JG30-KF-CM45* (11.72%), *Truepera* (7.23%), *Ornithinicoccus* (4.96%), and *Galbibacter* (4.55%) were dominant (Figure 5B).

### Comparison of bacterial composition among different bedding materials

The bacterial composition at different taxa levels among three bedding materials were further compared. At the phylum level, CF group had a significantly higher abundance of *Actinobacteria*, *Firmicutes*, *Corynebacteriaceae*, *Peptostreptococcaceae*, *Carnobacteriaceae*, *Staphylococcaceae*, and *Clostridiales* Family XI, and a significantly lower proportion of *Proteobacteria* and *Bacteroidetes* than other groups (p<0.05). There were no significant differences in the abundance of *Actinobacteria*, *Proteobacteria* and *Bacteroidetes* between CB and SB groups (p>0.05). The significantly higher abundance of *Chloroflexi*, *Deinococcus-Thermus*, *Flavobacteriaceae*, norank *JG30-KF-CM45*, and *Trueperaceae* were observed in the CB group compared with other groups (p< 0.05, Supplementary Table S1).

At the genus level, the abundances of *Ornithinicoccus*, *Ornithinimicrobium*, and *Iamia* genera were significantly higher in SB group than in CF and CB groups (p<0.05). Compared with SB or CB group, CF group showed a significantly higher relative abundance of *Corynebacterium*, *Corynebacterium_1*, *Dietzia*, *Micrococcus*, *Paeniclostridium*, unclassified *Peptostreptococcaceae*, unclassified *Carnobacteriaceae*, *Solibacillus*, *Aliicoccus*, *Atopostipes*, and *Facklamia* (p<0.05). The proportion of unclassified *JG30-KF-cM45*, *Truepera*, *Galbibacter*, unclassified *Flavobacteriaceae*, and *Simiduia* were significantly higher in CB group than in SB and CF groups (p<0.05; Supplementary Table S2). *Treponema* (0% to 0.07% in SB and 0% to 0.06% in CB, respectively) and *Treponema*-associated family *Spirochaetaceae* only presented in SB and CB samples (0% to 0.08% in SB and 0% to 0.06% in CB, respectively,) with very low abundances (Supplementary Figure S1).

### Correlation analysis between the bedding material bacteria and serum biomarkers of joint damage

**Figure 6:**
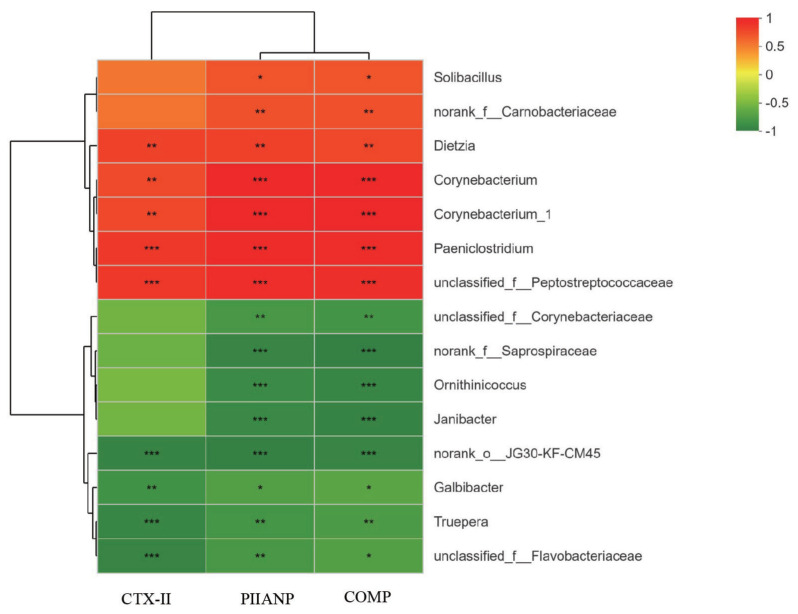
Pearson’s correlation heatmap showing the relationship between the abundance of top 15 genera in bedding material bacteria and serum biomarkers of joint damage. PIIANP, procollagen IIA N-terminal peptide; CTX-II, c-opeptide of type II collagen; COMP, cartilage oligomeric matrix protein. Significant differences were displayed with either * p<0.05, ** p<0.01, or *** p<0.001.

The potential functional relationships among bedding material bacteria and serum biomarkers of joint damage were investigated using Pearson’s correlation analysis. As displayed in Figure 6, The relative abundances of genera *Dietzia*, *Corynebacteriaceae*, *Corynebacterium*, *Corynebacterium_1* and *Paeniclostridium* were positively related to CTX-II, PIIANP, and COMP levels (p<0.01). In contrast, the relative abundances of *Galbibacter* and *Truepera* were inversely associated with CTX-II, PIIANP and COMP levels (p<0.05). The top 15 genera that were all significantly associated with PIIANP excretion were also associated with excretion of COMP.

## DISCUSSION

An unsuitable type of bedding materials was one of the important risk factors for lameness in dairy cows [8]. Cartilage was the most fragile among the joint tissues when exposed to damage factors, the imbalance between formation and degeneration of cartilage would lead to chronic joint inflammation. CTX-II accounts for 70% of cartilage composition and could be a specific indicator for cartilage destruction [18]. COMP is a non-collagen biomarker for early molecular changes in articular cartilage damages [19]. PIIANP can be used as one specific marker for type IIA procollagen synthesis in joint damage [20]. In this study, the CB housing system effectively improved animal welfare and joint health, as revealed by significantly lower concentration of CTX-II and PIIANP, gait performance and hock injuries score. These results were similar with the previous study that lame cows have increased serum CTX-II and COMP levels, the changes of joint damage biomarkers might have a correlation with oxidative status and mineral metabolism [21].

In addition to joint damage, DD is a polymicrobial foot disease causing lameness in dairy cows. DD-associated pathogens may be originated from the microbiome of cows’ oral cavity, gastrointestinal, and feces [9,10], based on which bedding materials may serve as the medium for transmitting DD-associated bacteria. Here, CB showed similar bacterial composition with SB while were structurally distinct from CF samples as revealed by Alpha and Beta diversity. The four dominant phyla *Actinobacteria*, *Firmicutes*, *Proteobacteria*, and *Bacteroidetes* found in three bedding materials were also reported to be abundant in bacteria of cows’ rumen content, feces, and healthy hoof skin [12,22]. In the current study, the relative abundances of *Chloroflexi* and *Deinococcus–Thermus* were significantly higher in SB and CB samples than in CF samples. Phyla *Chloroflexi* was reported to predominate in rumen bacteria of dairy cows and digesters treating sewage sludge cattle manure [22,23]. Another phyla, *Deinococcus–Thermus* decomposed high-molecular-weight organic matter such as starch, cellulose, proteins, xylan, and chitin [24].

One possible limitation of the present study was that hoof tissue bacteria were not assessed in the current study, which limited the relationship analysis between bedding bacterial composition and lameness. However, the pathogenic bacteria of DD have been well studied. *Spirochaetae* was previously described as the predominant pathogen in DD-associated lesions and *Treponemes* were actively expressing virulence factors at the site of infection [13]. In this study, phylum *Spirochaetae* and family *Spirochaetaceae*, with 0% to 0.08% relative abundance in different taxonomic levels, were only detected in SB and CB samples. Similary, *Treponema* was only detected in a small number of the SB and CB samples in this study (0.024% and 0.019%, respectively), this abundance was lower compared to the previous study [15], which reported an overall abundance of 0% to 0.6% for DD-associated treponemal amplicons in environmental slurry samples. The majority of *Treponema* could be further determined to those unclassified species which likely belonged to the nonpathogenic environmental members of the species in the current study. Our results could further support the hypothesis that *Treponeme* may potentially be relatively rare in the living environment of cows even the areas were highly frequently accessed by the dairy herd [15].

The genera norank *JG30-KF-cM45*, *Ornithinicoccus*, and *Truepera* were enriched in SB and CB samples. Norank *JG30-KF-cM45* and *Truepera* were identified as aerobic denitrifying bacteria, nitrogen-based pollutants could be converted to N_2_ and N_2_O through denitrifying bacteria and its related enzymes under aerobic conditions [25]. In CF samples, the dominant genera were *Corynebacterium* and *Corynebacterium_1* of family *Corynebacteriaceae*, along with *Paeniclostridium* and *Facklamia* of phylum *Firmicutes*. The SB and CB samples harbored a higher relative abundance of unclassified *Flavobacteriaceae* and *Galbibacter* and a similar proportion of *Flavobacterium* compared with CF samples. We also found that serum biomarkers of joint damage (CTX-II, PIIANP and COMP) were significantly positive associated with *Corynebacterium*, *Corynebacterium_1* and *Paeniclostridium*. As reported by previous study, the genera *Acinetobacter*, *Corynebacterium*, and *Flavobacterium* in ovine feet lesions were more closely linked to inflammation level than disease state [26]. *Corynebacterium* was dominant member of the skin microbiota in mice and humans and have been previously reported to promote activation of immune cells and control skin immunity and inflammation [27]. The members of *Paeniclostridium* were mainly associated with trauma, toxic shock, soft tissue skin infections [28], whereas *Facklamia* was gram-positive bacteria and regarded to be a potential pathogen of periprosthetic joint infection and clinical bloodstream infection [29].

*Corynebacteriaceae*, *Flavobacteriaceae*, *Peptostreptococcaceae*, and *Carnobacteriaceae* were the most abundant bacterial families in the three bedding materials. Most of these bacterial families were also present at relatively high abundances in interdigital skin samples from the healthy feet of dairy cattle or slurry samples from the dairy herd environment [13,15]. Moreover, in this study, *Clostridiales Family XI* was detected at significantly higher relative abundance in CF samples compared with SB and CB samples, and it was formerly found to be more abundant in skin lesions compared to healthy skin samples of cattle claws [2]. Our results also showed that the relative abundances of genera *Solibacillus* and *Clostridium sensu stricto 1* from the same phylum *Firmicutes* were significantly higher in CF samples than in other groups, those two genera were reported to be associated with gastrointestinal disease in human and cows [22,30].

In conclusion, the SB and CB are good alternatives in the dairy housing system in terms of joint health and lameness. The composition of bacterial communities significantly differed among bedding materials. However, Lameness-associated *Spirochaetaceae* and *Treponeme* were only detected in SB and CB samples with a low relative abundance, pathogens involved in the pathogenesis of DD may not be a part of microbiota in bedding materials of dairy cows.

## IMPLICATIONS

The bedding material was proven to be associated with animal welfare and lameness incidence in dairy cows. This study has detected the bacterial composition in different bedding materials, including sand bedding, concrete floor, and compost bedding. The results revealed that the pathogens involved in the pathogenesis of lameness may not be a part of microbiota in bedding materials, and that the compost bedding was an effective housing system to improve the performance and joint health of Holstein heifers.

## Figures and Tables

**Table 1:** Serum biomarkers of joint damage in dairy cows housed on different bedding materials

Items	Bedding materials			SEM	p-value

	Sand bedding	Concrete floor	Compost bedding		
PIIANP (ng/mL)	61.45^b^	79.29^a^	58.76^b^	2.817	0.001
CTX-II (ng/mL)	202.58^b^	223.62^a^	162.71^b^	10.076	0.048
COMP (ng/mL)	186.21	206.35	186.06	7.262	0.548

SEM, standard error of means; PIIANP, procollagen IIA N-terminal peptide; CTX-II, c-opeptide of type II collagen; COMP, cartilage oligomeric matrix protein.

a,b Mean in the same row with different superscripts represents a significant difference (p<0.05).

**Table 2:** Diversity indices and richness estimators of the bacteria in different bedding material

Items	Bedding materials			SEM	p-value

	Sand bedding	Concrete floor	Compost bedding		
Sequence number	50,445	46,865	49,250	1,168.034	0.487
OTU number	900.75^a^	463.50^b^	820.75^a^	59.249	<0.001
Shannon	5.14^a^	3.98^b^	5.03^a^	0.172	<0.001
Simpson	0.01^b^	0.05^a^	0.02^b^	0.005	<0.001
Ace	1,029.34^a^	524.11^c^	952.43^b^	67.958	<0.001
Chao	1,040.92^a^	527.13^c^	957.35^b^	69.144	<0.001
Coverage	0.995^b^	0.998^a^	0.998^b^	0.000	0.003

SEM, standard error of means; OTU, operational taxonomic units.

a–c Mean in the same row with different superscripts represents a significant difference (p<0.05).
