# Supplementary material for: Relationships among bedding materials, bedding bacterial composition and lameness in dairy cows
Source: Anim Biosci. 2020 Nov 3;34(9):1559–68. doi: 10.5713/ajas.20.0565 (PMC8495337; doi:10.5713/ajas.20.0565)
Supplement: Supplementary file 1 [file ajas-20-0565-suppl.pdf]

Supplemental material

Relationships among bedding materials, bedding bacterial composition and lameness in dairy cows

Han *Li*[1], Xiangming *Wang*[1], Yan *Wu*[1], Dingran *Zhang*[1], Hongyang *Xu*[1], Hongrun *Xu*[1], Xiaoguang *Xing*[1] and Zhili *Qi*[*1]

[*]**Corresponding author: Zhili Qi**

TEL:+86-13296621998, Fax: 027-87280408, E-mail: zhiliqi@mail.hzau.edu.cn

[1]Department of Animal Nutrition and Feed Science, College of Animal Science and Technology, Huazhong Agricultural University, 430070 Wuhan, China.

ORCID

Han *Li* https://orcid.org/0000-0001-9375-5944

Xiangming *Wang* https://orcid.org/0000-0002-1492-5915

Yan *Wu* https://orcid.org/0000-0002-7087-8850

Dingran *Zhang* https://orcid.org/0000-0003-4620-6293

Hongyang *Xu* https://orcid.org/0000-0001-8693-0978

Hongrun *Xu* https://orcid.org/0000-0002-4986-3793

Xiaoguang *Xing* https://orcid.org/0000-0001-6276-1081

Zhili *Qi* https://orcid.org/0000-0003-0112-0162

Table S1. Comparison of the bacterial phyla and family in all the samples from different bedding materials (the results were only presented for the phyla which relative abundance ≥ 0.08% and family which relative abundance ≥ 5% in at least one sample).

Taxa	Bedding materials			SEM[1]	p-value
	Sand bedding	Concrete floor	Compost bedding		
**Phylum**					
Actinobacteria	31.30^{b}	41.15^{a}	24.75^{b}	0.024	0.003
Firmicutes	13.10^{b}	50.75^{a}	7.71^{c}	0.058	<0.001
Proteobacteria	22.45^{a}	3.38^{b}	19.90^{a}	0.026	<0.001
Bacteroidetes	17.66^{a}	3.65^{b}	21.50^{a}	0.025	<0.001
Chloroflexi	9.84^{b}	0.12^{c}	14.87^{a}	0.019	<0.001
Deinococcus-Thermus	2.73^{b}	0^{b}	7.23^{a}	0.010	0.001
Saccharibacteria	0.83	0.78	1.23	0.001	0.260
Gemmatimonadetes	1.27^{a}	0^{b}	1.28^{a}	0.002	<0.001
Planctomycetes	0.22^{a}	0^{b}	0.30^{a}	0.001	0.023
Acidobacteria	0.08^{b}	0^{c}	0.25^{a}	0.000	<0.001
Fibrobacteres	0.07^{b}	0^{b}	0.18^{a}	0.000	0.005
bRC1	0.05^{b}	0^{b}	0.19^{a}	0.000	<0.001
Cyanobacteria	0.11	0.08	0.05	0.000	0.439
Verrucomicrobia	0.06	0.04	0.08	0.000	0.491
Parcubacteria	0.02^{b}	0^{b}	0.10^{a}	0.000	0.003
Tenericutes	0.02	0.03	0.04	0.000	0.269
Microgenomates	0^{b}	0^{b}	0.07^{a}	0.000	<0.001
Spirochaetae	0.04	0	0.03	0.000	0.182
**Family**					
Corynebacteriaceae	4.23^{b}	29.23^{a}	3.07^{b}	0.037	<0.001
Intrasporangiaceae	13.73^{a}	0.32^{c}	8.37^{b}	0.017	<0.001
Flavobacteriaceae	6.28^{b}	0.92^{c}	12.66^{a}	0.017	<0.001
norank *JG30-KF-CM45*	7.11^{b}	0.12^{c}	11.72^{a}	0.015	<0.001
Peptostreptococcaceae	3.80^{b}	9.73^{a}	2.11^{b}	0.010	<0.001
Carnobacteriaceae	1.28^{b}	7.92^{a}	0.97^{b}	0.011	0.001
Trueperaceae	2.73^{b}	0^{b}	7.23^{a}	0.010	0.002
Staphylococcaceae	1.05^{b}	6.50^{a}	0.15^{b}	0.011	0.003
Planococcaceae	0.80^{b}	5.65^{a}	0.76^{b}	0.010	0.006
Ruminococcaceae	1.08^{b}	5.06^{a}	0.47^{b}	0.008	0.009
Cellvibrionaceae	2.84^{a}	0^{b}	3.00^{a}	0.005	0.013

Clostridiales Family XI	0.42^{b}	4.43^{a}	0.06^{b}	0.006	0.048
Moraxellaceae	0.20	2.54	0.20	0.006	0.144

[1] SEM, standard error of means.

[a-c] Mean in the same row with different superscripts represents a significant difference ($p < 0.05$).

Table S2. Comparison of the top 30 genera in all the samples from different bedding materials.

Genus[1]	Bedding materials			SEM	*P*-Value
	Sand Bedding	Concrete floor	Compost bedding		
norank *JG30-KF-CM45*	7.11^{b}	0.12^{c}	11.72^{a}	0.015	<0.001
Corynebacterium	0.78^{b}	16.88^{a}	0.13^{b}	0.024	<0.001
Ornithinicoccus	7.38^{a}	0.15^{c}	4.96^{b}	0.010	<0.001
Corynebacterium_1	0.34^{b}	11.33^{a}	0.02^{b}	0.016	<0.001
Truepera	2.73^{b}	0.00^{b}	7.23^{a}	0.010	0.001
Paeniclostridium	2.11^{b}	5.66^{a}	1.18^{b}	0.006	<0.001
unclassified	3.11^{a}	1.03^{b}	2.92^{a}	0.004	0.033
unclassified	1.69^{b}	4.07^{a}	0.93^{b}	0.004	<0.001
Norank *Saprospiraceae*	3.63^{a}	0^{c}	2.66^{b}	0.005	<0.001
Galbibacter	1.59^{ab}	0^{b}	4.55^{a}	0.008	0.030
Unclassified	1.49^{b}	0.14^{b}	4.49^{a}	0.006	0.001
Janibacter	3.43^{a}	0.06^{b}	2.48^{a}	0.005	<0.001
Dietzia	1.73^{b}	3.30^{a}	0.87^{b}	0.004	0.003
norank *Carnobacteriaceae*	0.85^{b}	3.56^{a}	0.79^{b}	0.005	0.039
Solibacillus	0.25^{b}	4.61^{a}	0.08^{b}	0.008	0.026
Pseudomonas	2.85^{a}	0.14^{b}	1.93^{a}	0.004	0.011
Aliicoccus	0.57^{b}	3.42^{a}	0.00^{b}	0.005	0.002
Ornithinimicrobium	2.91^{a}	0.11^{c}	0.92^{b}	0.004	<0.001
Atopostipes	0.34^{b}	3.38^{a}	0.16^{b}	0.005	0.001
Micrococcus	0.21^{b}	3.05^{a}	0.07^{b}	0.004	<0.001
Iamia	2.18^{a}	0.01^{c}	1.07^{b}	0.003	<0.001
norank *Anaerolineaceae*	1.64^{a}	0^{b}	1.49^{a}	0.003	0.003
Simiduia	1.26a^{b}	0^{b}	1.76^{a}	0.003	0.0036
Facklamia	0.16^{b}	2.81^{a}	0.03^{b}	0.004	<0.001
unclassified	2.10^{a}	0.05^{c}	0.81^{b}	0.003	<0.001
Acinetobacter	0.20	2.54	0.19	0.006	0.143
Turicibacter	0.84^{b}	1.56^{a}	0.46^{c}	0.002	<0.001
norank *Saccharibacteria*	0.78	0.73	1.19	0.001	0.217
Marinobacter	1.49^{a}	0^{b}	0.86^{a}	0.002	0.011
Clostridium sensu stricto_1	0.83^{a}	1.04^{a}	0.33^{b}	0.001	0.002

[1] Some taxa have no scientific names in the taxonomic database, marked with norank. Some taxa pedigrees are below the confidence threshold, no classification information is obtained, marked with unclassified.

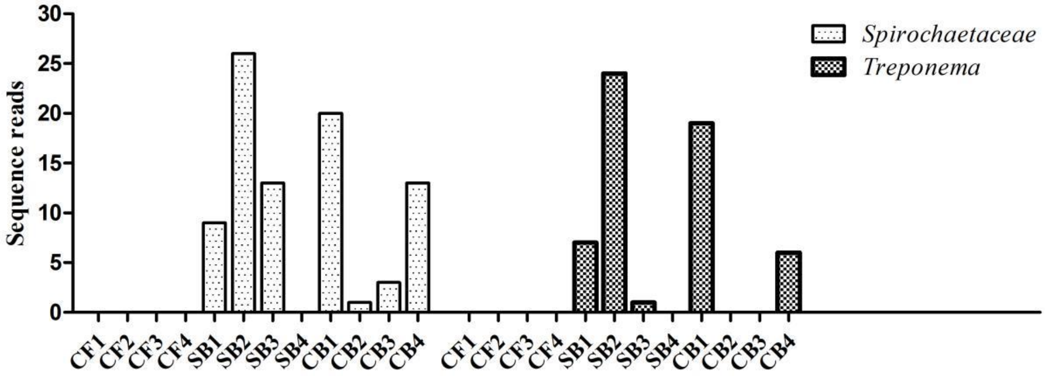

Figure S1. The abundance of lameness-associated digital dermatitis family *Spirochaetaceae* and genus *Treponema* in the samples from bedding materials. SB, sand bedding; CF, concrete floor; CB, compost bedding.
